# Merkel Cell Polyomavirus and DNA Damage Response (DDR): Transcriptional Analysis of DDR Pathways in the Context of Merkel Cell Carcinoma

**DOI:** 10.3390/cancers18101592

**Published:** 2026-05-14

**Authors:** Sara Messina, Domenico Mallardo, Amedeo Ferlosio, Lucia Festino, Claudia Trojaniello, Rossella Di Trolio, Marco Ciotti, Paolo Antonio Ascierto, Valeria Pietropaolo, Sara Passerini

**Affiliations:** 1 Department of Public Health and Infectious Diseases, Sapienza University of Rome, 00185 Rome, Italy; sara.messina@uniroma1.it; 2 Unit of Melanoma, Cancer Immunotherapy and Development Therapeutics, Istituto Nazionale Tumori IRCCS “Fondazione G. Pascale”, 80131 Naples, Italy; d.mallardo@istitutotumori.na.it (D.M.); l.festino@istitutotumori.na.it (L.F.); claudia.trojaniello@istitutotumori.na.it (C.T.); r.ditrolio@istitutotumori.na.it (R.D.T.); p.ascierto@istitutotumori.na.it (P.A.A.); 3 Anatomic Pathology, Department of Biomedicine and Prevention, Tor Vergata University of Rome, 00133 Rome, Italy; amedeo.ferlosio@ptvonline.it; 4 Virology Unit, Polyclinic Tor Vergata Foundation, 00133 Rome, Italy; marco.ciotti@ptvonline.it; 5 Dipartimento di Neuroscienze e Scienze Riproduttive ed Odontostomatologiche, Università degli Studi di Napoli “Federico II”, 80125 Naples, Italy

**Keywords:** MCPyV, MCC, DDR, *ATM*, *ATR*, *p53*

## Abstract

**Background/Objectives:** Merkel cell polyomavirus (MCPyV) is a ubiquitous virus strictly associated with Merkel cell carcinoma (MCC), a rare and aggressive skin cancer. MCPyV oncogenic properties are associated mainly with early protein expression, integration, and LT truncation. MCPyV can also interact with DNA Damage Response (DDR) mechanisms, contributing to oncogenesis and tumor progression. In this work, we investigated the correlation between MCPyV and MCC and evaluated the mRNA expression profiles of DDR genes in virus-positive and -negative tumors. **Methods:** A total of 19 formalin-fixed paraffin-embedded biopsies were acquired from patients diagnosed with MCC. After DNA and RNA extraction, the DNA was used for MCPyV detection via qPCR and for sequencing analysis of the early, late, and non-coding control viral regions and the extracted RNA was used for MCPyV transcripts, miRNA detection and for the evaluation of several DDR genes expression such as *ATM*, *ATR*, *CHK1*, *CHK2*, *H2AX*, *Rad51*, *p53*, and *p21*, in MCPyV-positive and -negative samples via reverse transcription, PCR, and qPCR. **Results:** MCPyV presence was detected in 11/19 samples, all characterized by viral integration, LT truncation, and early region expression only. Furthermore, higher mRNA levels of DDR genes were observed in MCPyV-positive tumors compared with the negative ones. **Conclusions:** Our findings support the role of MCPyV in MCC formation and suggest its involvement in the transcriptional regulation of DDR genes, which may influence tumor progression. Understanding the molecular interplay between MCPyV and the DDR may guide future research into plausible novel diagnostic and therapeutic strategies for virus-induced tumors.

## Simple Summary

Merkel cell carcinoma (MCC) is a highly aggressive cancer primarily caused by Merkel cell polyomavirus (MCPyV), which interferes with multiple cellular pathways, including the DNA Damage Response (DDR). The aim of this study was to analyze the presence and the molecular status of MCPyV in MCC samples and to assess the viral influence on the DDR pathway. We confirmed the viral etiology of MCC tumors and observed overexpression of several DDR genes including *ATM*, *ATR*, *CHK1*, *CHK2*, *H2AX*, *Rad51*, *p53*, and *p21*. Molecular differences between virus-positive and virus-negative MCCs could serve as diagnostic markers as well as allow to design specific therapeutic strategies for virus-positive MCC cancers.

## 1. Introduction

The cellular system is exposed daily to endogenous and exogenous factors responsible for DNA lesions, which may compromise cellular functions [1]. In this framework, organisms have developed a complex machinery known as DNA Damage Response (DDR), involving several pathways that mediate cell cycle arrest to allow DNA repair or activate processes such as cell death or senescence, when the extent of the damage exceeds the cell’s repair capacity [2]. To overcome the wide variety of DNA lesions, cells have evolved multiple distinct DNA repair mechanisms [3]. Key components in DDR signaling are Ataxia-Telangiectasia Mutated (ATM) and Ataxia telangiectasia and Rad3-related (ATR), two protein kinases that recognize double-strand breaks (DSBs) and single-strand breaks (SSBs) respectively, and mediate the phosphorylation of several target proteins, including checkpoint kinase (CHK) 1, CHK2, and p53 [4,5]. Specifically, following DSBs, the MRN complex recruits ATM to the site of DNA damage through interaction with the NSB1 protein [6,7]. Once activated, ATM mediates phosphorylation of downstream proteins such as CHK2 and p53, leading to cell cycle arrest [3,7]. In parallel, ATR responds to SSBs and phosphorylates CHK1, which in turn phosphorylates several target proteins, many of them overlapping with CHK2 substrates [5]. For instance, p53 can be phosphorylated by either CHK1 or CHK2, thereby promoting its function as a transcription factor to induce downstream gene expression, including *p21* [5].

Moreover, both activated ATM and ATR can phosphorylate the histone variant H2AX, whose phosphorylated form, γH2AX, is recognized as a general marker of DNA damage [8].

Since these pathways act collectively to preserve genomic integrity and stability, their alteration may lead to the accumulation of DNA lesions, promoting tumorigenesis [9]. Among external factors able to trigger DDR signaling, viral infections have also been recognized [1]. Notably, DDR processes are of particular interest in the case of oncogenic viruses, including Epstein–Barr virus [10], hepatitis B and C viruses [11] and Human Papillomavirus [12].

Another oncogenic virus involved in DDR manipulation is Merkel cell polyomavirus (MCPyV), a small DNA virus belonging to the *Polyomaviridae* family, first discovered in 2008 [13]. The viral genome is functionally divided into an early region encoding for Large T (LT), small T (sT) and 57 kT antigens (Ag) and alternative LT open reading frame (ALTO), involved in viral replication [14], and a late region encoding for structural proteins viral protein (VP) 1 and 2, and for two microRNAs (miRNAs), MCV-miR-M1-5p and MCV-miR-M1-3p, plausibly involved in the regulation of viral replication [15]. Interposed between the two coding regions is the Non-Coding Control Region (NCCR), which contains the origin of viral replication and bidirectional promoters for viral transcription [16,17].

MCPyV is widely diffused in the general population, mostly with asymptomatic infections [17,18]. However, it is also recognized as an oncogenic virus, due to its association with Merkel cell carcinoma (MCC), a rare and aggressive neuroendocrine skin cancer [13,19]. Approximately 80% of MCC cases are caused by MCPyV infection, while the remaining 20% are associated with ultraviolet (UV) exposure [20]. Specifically, virus-positive tumors exhibit clonal integration of viral DNA into the host genome [13], resulting in the expression of a truncated form of the LT protein (tLT), which loses the replicative function but maintains oncogenic properties, thus leading to increased cell proliferation and tumor development [21]. MCPyV oncogenic properties are strictly related to the capability of early proteins LT and sT to manipulate cellular processes by interacting with host proteins [22,23,24], including the DDR machinery [25]. Indeed, previous studies investigating the interplay between MCPyV and DDR revealed an activation of ATR/CHK1 and ATM/CHK2 pathways in MCPyV-positive cells, thereby promoting the phosphorylation and stabilization of the p53 protein, associated with cell cycle arrest and apoptosis [26]. Consistent with this hypothesis, Tsang et al. reported the accumulation of DDR components, including ATR, CHK1, γH2AX, and CHK2, in LT-positive nuclear foci [27]. Although some studies reported a modulation of DDR activity by LT alone, a contribution of sT in stimulating the DDR machinery has also been observed. Indeed, the expression of sT in MCPyV-positive MCC cells was found sufficient to induce the DDR signaling, leading to increased γH2AX levels and activation of the ATM pathway [28]. Notably, MCPyV may trigger DDR to promote viral replication, while also exploiting DDR activity to phosphorylate LT at Ser816, a modification associated with inhibition of cell proliferation [29]. Finally, our recent research reported overexpression of DDR genes, including *ATM*, *ATR*, *CHK1*, and *CHK2*, in MCPyV-positive MCC, suggesting a plausible role of the virus in manipulating DDR at the transcriptional level [30]. Considering these findings, our study aimed to investigate the expression profile of selected DDR genes in MCC tissues stratified by viral status.

This approach may help characterize MCPyV-associated differences in DDR gene transcriptional patterns and facilitate the identification of candidate therapeutic targets and diagnostic or prognostic biomarkers.

## 2. Materials and Methods

### 2.1. Sample Collection

Nineteen formalin-fixed paraffin-embedded (FFPE) primary tumor samples were collected from MCC patients. Specifically, ten cases (MCC1-10) (4 males, 6 females, mean age ± standard dev. 70 ± 7.58 years) were obtained from the unit of Melanoma, Cancer Immunotherapy and Development Therapeutics, Istituto Nazionale Tumori IRCCS Fondazione “G. Pascale” (Naples, Italy), and nine samples (MCC11-19) (9 males, mean age ± standard dev. 72 ± 12.85 years) were collected from the Dermatology Clinic of Tor Vergata University Hospital (Rome, Italy). All procedures were performed in accordance with the Declaration of Helsinki and were approved by the Ethics Committees of the Istituto Nazionale Tumori (Naples, Italy) (Protocol number: 32/22oss) and the Tor Vergata University (Rome, Italy) (Protocol number: 0015440/2019).

### 2.2. Histological Diagnosis

**Figure 1:**
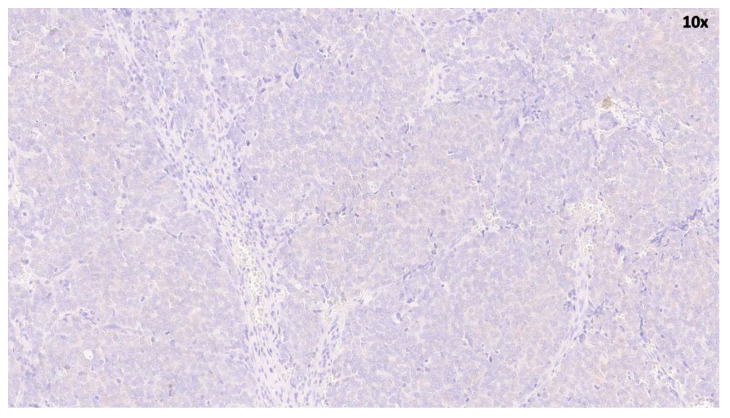
Low magnification (×10) of MCC section stained with hematoxylin and eosin. Typical features of MCC are evident, including the high nuclear/cytosol ratio and hyperchromatic nuclei.

The histological diagnosis of MCC was established based on the observation of an expansive, nodular, or diffusely infiltrating tumor located in the dermis of subcutis, where small round blue cells with scant cytoplasm and a high nuclear/cytoplasmic ratio. Nuclei exhibit indistinct nucleoli and granular chromatin with a characteristic salt-and-pepper pattern (Figure 1). Furthermore, conspicuous mitoses and apoptotic bodies were observed. The examined tissues revealed positivity for the epithelial marker CK20 with a perinuclear dot-like pattern, CAM5.2, and pan-cytokeratins, as well as for neuroendocrine markers, including chromogranin A and synaptophysin. Negative results were observed for K7, TTF1, LCA, S100, and cdx2.

### 2.3. DNA Extraction

FFPE tissues were deparaffinized with xylene, and DNA was subsequently extracted using the Quick DNA FFPE Miniprep Kit (Zymo Research, Irvine, CA, USA). Total DNA was eluted in 50 µL and evaluated for suitability for polymerase chain reaction (PCR) by amplifying the *β-globin* gene sequences [31].

### 2.4. Detection of MCPyV DNA by Polymerase Chain Reaction (qPCR)

MCPyV detection and quantification were performed by quantitative PCR (qPCR) using primers for the sT sequence, as previously described [32]. Viral load was evaluated using standard curves generated from a ten-fold serial dilution of the pMCV-R17a plasmid (#24729, Addgene, Cambridge, MA, USA), which contains the complete viral genome, and results were expressed as copies per milliliter (copies/mL), based on the fixed elution volume used during extraction [32,33]. Each qPCR reaction included the plasmid pMCV-R17a as a positive control, and nuclease-free water was used as a negative control.

### 2.5. PCR for LTAg, VP1 and NCCR Regions and Sequencing

Samples that tested positive for MCPyV DNA were subsequently subjected to PCR targeting viral regions corresponding to LTAg (LT1, LT3), NCCR, and VP1 [13,31]. PCR assays included the pMCV-R17a as a positive control in each run, whereas nuclease-free water served as a negative control. PCR products were visualized under UV light through electrophoresis on a 2% agarose gel using SafeView reagent (Applied Biological Materials, Vancouver, BC, Canada). Furthermore, to detect NCCR and VP1 variation, the amplified products were purified using the miPCR purification kit (Metabion, Planegg, Germany) according to the manufacturer’s instructions and sequenced (Bio-Fab Research, Rome, Italy). The obtained VP1 and NCCR sequences were compared with the reference strain deposited in GeneBank, MCC350:EU375803. Then, sequence alignment was performed with Clustal W2 on the European Molecular Biology Laboratory–European Bioinformatics Institute (EMBL–EBI) website (http://www.ebi.ac.uk/Tools/msa/clustalw2/ accessed on 2 March 2026).

### 2.6. Sequencing Analysis of LTAg

For LTAg sequencing (151-3102 nucleotide position, GenBank strain EU375803), six primer sets were employed to perform PCR assays [31], followed by sequencing (Bio-Fab Research, Rome, Italy).

### 2.7. Analysis of Viral Integration Sites

MCPyV genome integration was observed using the detection of the integrated papilloma sequence (DIPS)–PCR technique, which allows the amplification and detection of the junction between viral and host DNA [33,34]. In detail, the eluted DNA was fragmented by the TaqI restriction enzyme, generating several fragments that were further ligated with enzyme-specific adaptors. The ligated fragment undergoes PCR amplification using primers that recognize viral and adaptor sequences. Sequencing analysis was conducted, and the integration sites were established using the Basic Local Alignment Search Tool (BLAST + 2.17.0) for genomic localization.

### 2.8. RNA Extraction and Analysis

Total RNA was extracted from FFPE biopsies with the FFPE RNA purification kit (Norgen, Thorold, ON, Canada) and eluted in 50 μL. RNA quality and concentration were assessed using spectrophotometric reading (Take3 module of the Microplate Reader BioTek SynergyHT, BioTek Instruments, Winooski, VT, USA). All samples met the predefined quality thresholds (A260/230 ≥ 1.8) and were included in analysis. Reverse transcription (RT) was performed using the SensiFAST cDNA Synthesis kit (Meridian Bioscience, Cincinnati, OH, USA) and cDNA quality was assessed through the amplification of *β-globin* sequences [31]. Therefore, an aliquot of cDNA was used as a template to analyze LTAg and VP1 gene expression by PCR with specific primers [31,33]. Each run included positive and negative controls. Moreover, no-RT controls were included to exclude genomic DNA contamination.

### 2.9. Viral miRNA Analysis

For MCPyV-encoded miRNA detection, the Applied Biosystems™ TaqMan™ MicroRNA Assays (Thermo Fisher Scientific, Waltham, MA, USA) were used. Specifically, an aliquot of cDNA was subjected to qPCR using mcv-miR-M1-5p (ID006356). Furthermore, RNU6B (ID001093) was selected as an endogenous control.

### 2.10. Detection of LT by Immunohistochemistry

Among the collected samples, 10 exhibited adequate material for the detection of MCPyV Large T antigen by immunohistochemistry on FFPE tissue sections. Staining was performed using the Leica BOND automated staining platform (Leica Biosystems, Wetzlar, Germany). Following antigen retrieval, sections were incubated with a mouse monoclonal antibody directed against MCPyV Large T antigen, clone CM2B4, applied at a dilution of 1:100. Immunoreactivity was visualized using a polymer-based detection system according to the manufacturer’s instructions. LT expression was assessed based on nuclear staining in tumor cells. Cases were considered positive when distinct nuclear immunoreactivity was observed in tumor cells, whereas a complete absence of nuclear staining was interpreted as negative.

### 2.11. Analysis of DDR Genes’ Expression

RT-qPCR was performed to measure relative levels of *ATM*, *ATR*, *CHK1*, *CHK2*, *H2AX*, *Rad51*, *p53*, and *p21* transcripts [30,35,36,37]. The *β-globin* gene was used as a reference gene, selected based on its previously validated use in MCC tissues [30] and its consistent amplification across all analyzed specimens. mRNA relative levels were calculated by the comparative ΔCt method and reported as 2^−^^ΔCt^.

### 2.12. Statistical Analysis

For statistical analysis, results from three independent experiments were expressed as the mean ± standard deviation. Both viral load and gene expression were tested using the Shapiro–Wilk test to assess normal distribution. The relative expression of DDR genes in MCPyV-positive and -negative groups was compared by Student’s *t*-test with Welch’s correction when necessary. *p* < 0.05 was considered statistically significant. Outlier analysis was also performed using the ROUT method (Q = 1%).

## 3. Results

### 3.1. Detection of MCPyV DNA by qPCR and PCR

**Figure 2:**
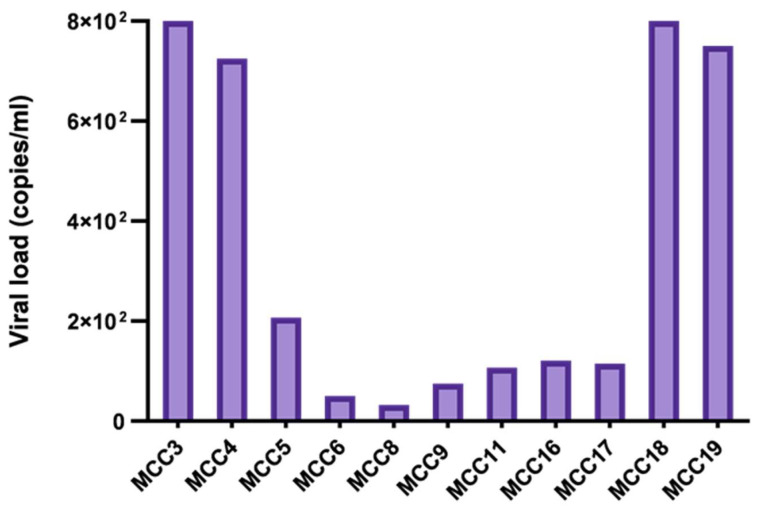
MCPyV load in virus-positive tissues from MCC patients.

MCPyV DNA was detected in 11/19 (57.9%) patients, including 9/13 (69.2%) males and 2/6 (33.3%) females. All samples were primary tumors and displayed a mean viral load of 3.4 × 10^2^ copies/mL (Figure 2, Table 1). The same samples also tested positive for LT1 and LT3, as well as for VP1 and NCCR amplification (Table 1).

### 3.2. Analysis of Viral Sequences

When analyzing NCCR and VP1 regions, canonical sequences were detected in all virus-positive samples, revealing a high degree of homology with the reference strain MCC350 (Table 1). Moreover, truncated LTAg were observed due to frameshift mutations generating stop codons, which preserve the pRb-binding motif LFCDE but eliminate the helicase domain (Table 1).

### 3.3. Viral Integration

Viral integration sites were identified in all virus-positive tumors. Specifically, viral junction sites varied from nuclear positions 1399 to 2952, whereas cellular junctions were mainly observed at chromosomes 5 and 6, except for two samples (MCC6 and MCC8), where integration was detected on chromosomes 4 and 1 (Table 1).

### 3.4. Expression of LT and VP1 Transcripts

MCC-positive tissues were further subjected to RT-PCR analysis for MCPyV *LTAg* and *VP1* genes. In all samples, the *LTAg* expression has been noted, whereas the *VP1* gene was not detected in any tumor (Table 2).

### 3.5. Viral miRNA Expression

RNA extracted from virus-positive MCCs was also tested for mcv-miR-M1-5p; however, none of the samples showed positivity to MCPyV-encoded miRNA (Table 2).

### 3.6. Detection of LT Protein

**Figure 3:**
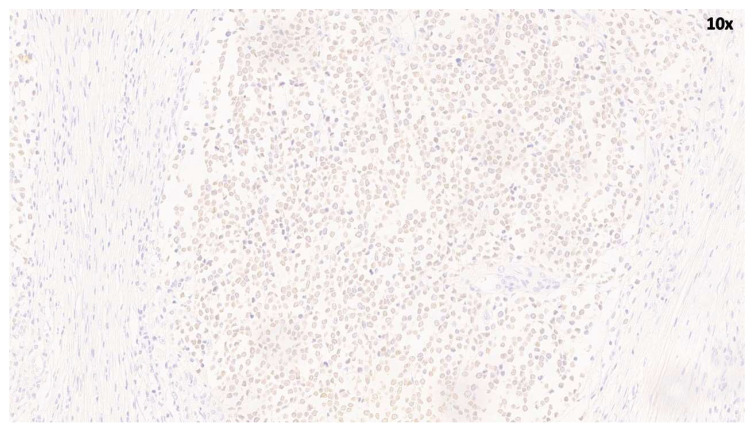
Immunohistochemical analysis of MCC showing nuclear positivity for MCPyV.

Immunohistochemical analysis was performed on 10 cases (MCC1–MCC10), including six PCR-positive and four PCR-negative cases. MCPyV LT nuclear expression was reported in five out of 10 MCC samples (Table 1). LT-positive cases showed strong and diffuse nuclear staining in tumor cells. In contrast, no immunoreactivity was observed in the remaining five MCPyV-negative samples, which were completely devoid of staining. These findings are consistent with the presence of MCPyV in a subset of MCC tumors (Figure 3).

### 3.7. Relative Expression of DDR Genes

**Figure 4:**
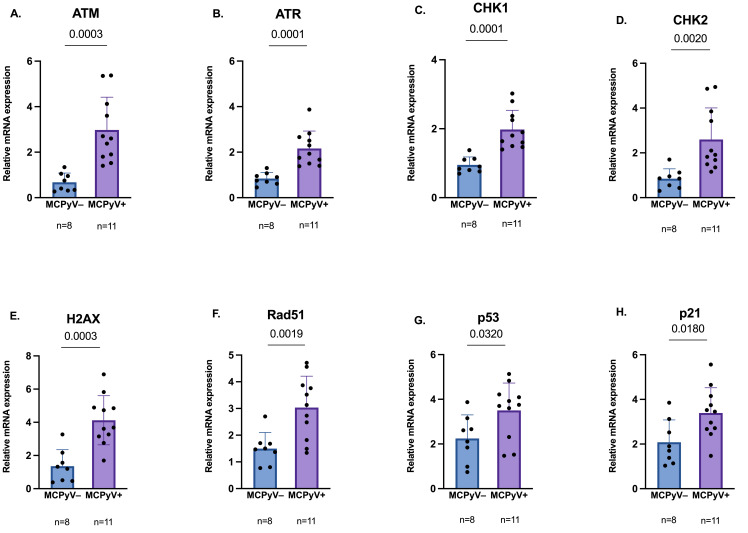
Relative expression of DDR genes in MCPyV-positive (n = 11) and -negative (n = 8) MCC samples (Panel (**A**), *ATM*; Panel (**B**), *ATR*; Panel (**C**), *CHK1*; Panel (**D**), *CHK2*; Panel (**E**), *H2AX*; Panel (**F**), *Rad51*; Panel (**G**), *p53*; Panel (**H**), *p21*). Gene expression values exhibited a normal distribution (Shapiro–Wilk test; *p* > 0.05) and mean values of positive and negative MCCs groups were compared using an independent *t*-test with Welch’s correction.

Among positive and negative MCC biopsies, a significant difference in expression levels of several DDR genes, such as *H2AX*, *ATM*, and *ATR*, and downstream effectors *CHK1*, *CHK2*, *Rad51*, *p53*, and *p21* was observed (*p* < 0.05) (Figure 4).

## 4. Discussion

Host cells often incur DNA damage as a result of viral infection. Indeed, several viruses can induce genetic instability through DNA damage during viral replication. Moreover, some viruses can directly interfere with components of the DDR, an intricate network of proteins evolved to detect and repair DNA lesions [25,38]. Among them, MCPyV was also found to interact and regulate DDR proteins, likely promoting tolerance to viral replication in MCC [26,27,39]. Despite these observations, limited data from MCC cases do not yet establish a direct correlation between MCPyV and DDR manipulation.

In the current study, we investigated MCPyV prevalence in a cohort of MCC specimens obtained from two Italian hospitals, revealing MCPyV DNA in approximately 58% of samples and thus confirming the viral etiology of MCC formation [13]. Moreover, viral integration and LTAg truncation, as well as lack of late gene expression were reported, strongly supporting these molecular features as hallmarks of virus-mediated oncogenesis [13,19].

Specifically, numerous integration sites were detected without evidence of specific preferential sites in the MCPyV genome, whereas in the host genome, the long arm of chromosome 5 was confirmed as the most frequent localization [40,41]. Sequencing analysis showed canonical NCCR and VP1, reinforcing that mutations within these regions may not be involved in tumorigenesis [14,33].

Consistent with previous research [33], viral miRNAs were not detectable, leaving their biological relevance in cancer still unclear [42]. However, their absence, likely due to MCPyV miRNAs being expressed during the lytic infection together with late genes, combined with the lack of VP1 expression [15], indicates that viral integration disrupts transcription of the late region [17].

Once viral prevalence and molecular state in MCC specimens were assessed, the current study aimed to explore the DDR pathway at the RNA level. Consistent with our previous research suggesting a virus-mediated transcriptional activation of DDR [30], MCC tumors displayed distinct expression profiles of DDR genes, depending on viral status, with MCPyV-positive samples reporting higher *ATM*, *ATR*, *CHK1* and *CHK2* mRNA levels compared to negative tissues. Furthermore, an overexpression of *H2AX*, *p53*, *p21* and *Rad51* was reported, reinforcing the possibility that viral infection may induce changes in DDR components already at the RNA level [30,43]. The observed upregulation of *ATM* and *ATR*, alongside their downstream effectors *CHK2* and *CHK1*, suggests that MCPyV induces multiple forms of genomic stress, mainly due to viral interference with host DNA replication and to DNA damage caused by viral integration [30]. Moreover, the concurrent activation of CHK1 and CHK2 is consistent with a plausible role of the virus in manipulating cell cycle checkpoints to maintain the host cell in a state amenable to viral replication [27,44]. Therefore, like other oncogenic viruses, MCPyV infection may induce replication stress and DDR, but simultaneously exploits this signaling to favor its own replication [45]. This fine-tuning between DDR activation and checkpoint modulation may facilitate viral replication and persistence, which can lead to genomic instability and contribute to tumorigenesis [30,46].

In addition, the overexpression of *H2AX* mRNA may be linked with increased DNA repair in virus-positive tumors [47]. Indeed, although γH2AX is typically discussed as a post-translational marker of DNA damage, higher *H2AX* mRNA levels may reflect chronic DDR activation upon viral infection, consistent with in vitro studies showing H2AX phosphorylation and ATM activation by MCPyV sT [28]. Elevated *Rad51* expression further suggests enhanced homologous recombination repair in MCPyV-positive MCC [46]. Similar observations have been reported in HPV-associated malignancies, where increased *Rad51* mRNA levels were detected [48].

In MCPyV-positive MCC, increased expression of *p53* and *p21* may reflect an adaptive response to DNA damage rather than effective tumor suppression [21]. In contrast, MCPyV-negative tumors, frequently driven by UV-induced mutation signatures and TP53 inactivation [49], may lack the coordinated activation of DDR pathways. However, despite reaching statistical significance, differences in *p53* and *p21* expression may have limited biological relevance given the similar distribution patterns in virus-positive and virus-negative MCCs.

Notably, these observations are limited to transcriptional changes and do not necessarily imply functional activation of the DDR signaling in vivo.

Beyond transcriptional regulation, epigenetic mechanisms may contribute to the sustained high expression of DDR genes in MCPyV-positive tumors. Consistent with this hypothesis, Cheng *and colleagues* reported the role of sT in binding to L-MYC to recruit the EP400 histone acetyltransferase and chromatin remodeling complex [50]. In addition, differential methylation patterns were observed between virus-positive and virus-negative MCC, suggesting that viral status may influence genome methylation [51,52]. Moreover, recent evidence suggests that MCC oncogenesis involves virus-induced changes in chromatin state and histone methylation, potentially facilitating persistent DDR gene transcription and modifying checkpoint pathways [53]. Therefore, viral components may interact with and recruit chromatin remodeling complexes, which could contribute to epigenetic modulation of DDR components. However, further studies investigating the methylation status of DDR genes are needed to confirm this hypothesis, which remains speculative.

Despite providing novel insights into the differential expression of DDR genes in MCPyV-positive and -negative MCC, our study has several limitations. The analysis based on RNA expression data may not fully reflect protein abundance or functional activity of DDR components, particularly in the case of post-translational modifications such as phosphorylation of downstream kinases CHK1 and CHK2 or γH2AX foci formation.

Moreover, the study is observational and cannot establish causality between viral status and DDR activation. Finally, the limited sample size, reflecting the rarity of MCC and the lack of a control group, including normal adjacent skin from patients and healthy controls, may not fully represent DDR modulation across tumors.

Overall, our findings showed an overexpression of DDR genes in virus-positive tumors. Since DDR genes, including *ATM*, *CHK2*, *Rad51* and *H2AX*, were highlighted as potential markers for predicting treatment response in human tumors [36,47,54], monitoring the expression levels of DDR genes may be a candidate transcriptional signature for MCC tumors; however, any prognostic or predictive relevance remains speculative in the absence of patient outcome and requires further validation in a clinically annotated cohort.

## 5. Conclusions

In conclusion, the current hypothesis-generating study reports transcriptional upregulation of DDR genes in MCPyV-positive MCC tumors, suggesting viral modulation of host repair mechanisms. In light of these primarily descriptive results, additional research, including protein analysis, functional assays and clinical correlation, will be essential to validate these observations and to further explore their clinical relevance in this aggressive malignancy.

## Figures and Tables

**Table 1:** Summary of MCPyV viral load and molecular state in virus-positive MCCs.

Sample ID	Sex	Viral Load	LTAg		NCCR	VP1	LTAg	Integration Sites		IHC
		(Copies/mL)	LT1	LT3				Viral Junction	Cellular Junction	LT
MCC3	F	8 × 10^2^	+	+	canonical	canonical	truncated	1952-3′	6p22.3	+
MCC4	M	7.25 × 10^2^	+	+	canonical	canonical	truncated	2234-3′	6p12.3	+
MCC5	M	2.07 × 10^2^	+	+	canonical	canonical	truncated	1776-3′	6q24.1	+
MCC6	F	5 × 10^1^	+	+	canonical	canonical	truncated	2351-3′	4p15.2	−
MCC8	M	3.2 × 10^1^	+	+	canonical	canonical	truncated	1522-3′	1p36.2	+
MCC9	M	7.5 × 10^1^	+	+	canonical	canonical	truncated	2952-3′	5q35.1	+
MCC11	M	1.07 × 10^2^	+	+	canonical	canonical	truncated	2597-3′	5q11.2	NA
MCC16	M	1.21 × 10^2^	+	+	canonical	canonical	truncated	1399-3′	5q11.2	NA
MCC17	M	1.15 × 10^2^	+	+	canonical	canonical	truncated	2843-3′	5q11.2	NA
MCC18	M	8 × 10^2^	+	+	canonical	canonical	truncated	5′-2738	5q23.1	NA
MCC19	M	7.5 × 10^2^	+	+	canonical	canonical	truncated	5′-2597	5q11.2	NA

NA: not available.

**Table 2:** MCPyV *LTAg* and *VP1* transcripts and miRNAs in MCC cases.

Sample ID	*LTAg*	*VP1*	miR-5p
MCC3	+	−	−
MCC4	+	−	−
MCC5	+	−	−
MCC6	+	−	−
MCC8	+	−	−
MCC9	+	−	−
MCC11	+	−	−
MCC16	+	−	−
MCC17	+	−	−
MCC18	+	−	−
MCC19	+	−	−

## Data Availability

Data is contained within the article.

## Abbreviations

The following abbreviations are used in this manuscript:

DDR	DNA damage response
ATM	ataxia-telangiectasia mutated
ATR	ataxia telangiectasia and Rad3-related
DSB	double-strand break
SSBs	single-strand break
CHK	checkpoint kinase
MCPyV	Merkel cell polyomavirus
LT	large T
sT	small T
Ag	antigen
ALTO	alternative LT open reading frame
VP	viral protein
miRNA	microRNA
NCCR	non-coding control region
MCC	Merkel cell carcinoma
UV	ultraviolet
tLT	truncated LT
Ser	serine
FFPE	formalin-fixed paraffin-embedded
PCR	polymerase chain reaction
qPCR	quantitative PCR
DIPS	detection of the integrated papilloma sequence
RT	reverse-transcribed
